# Spontaneous blinking and brain health in aging: Large-scale evaluation of blink-related oscillations across the lifespan

**DOI:** 10.3389/fnagi.2024.1473178

**Published:** 2025-01-07

**Authors:** Sujoy Ghosh Hajra, Jed A. Meltzer, Prerana Keerthi, Chloe Pappas, Allison B. Sekuler, Cam-CAN Group, Careesa Chang Liu

**Affiliations:** ^1^Department of Biomedical Engineering and Science, Florida Institute of Technology, Melbourne, FL, United States; ^2^Rotman Research Institute, Baycrest Health Sciences, Toronto, ON, Canada; ^3^School of Biomedical Engineering, McMaster University, Hamilton, ON, Canada; ^4^School of Computer Science, McGill University, Hamilton, ON, Canada

**Keywords:** aging, blinking, neurophysiology and brainwaves, blink-related oscillations (BROs), magnetoencephalography (MEG), precuneus

## Abstract

Blink-related oscillations (BROs) are newly discovered neurophysiological brainwave responses associated with spontaneous blinking, and represent environmental monitoring and awareness processes as the brain evaluates new visual information appearing after eye re-opening. BRO responses have been demonstrated in healthy young adults across multiple task states and are modulated by both task and environmental factors, but little is known about this phenomenon in aging. To address this, we undertook the first large-scale evaluation of BRO responses in healthy aging using the Cambridge Centre for Aging and Neuroscience (Cam-CAN) repository, which contains magnetoencephalography (MEG) data from a large sample (*N* = 457) of healthy adults across a broad age range (18–88) during the performance of a simple target detection task. The results showed that BRO responses were present in all age groups, and the associated effects exhibited significant age-related modulations comprising an increase in sensor-level global field power (GFP) and source-level theta and alpha spectral power within the bilateral precuneus. Additionally, the extent of cortical activations also showed an inverted-U relationship with age, consistent with neurocompensation with aging. Crucially, these age-related differences were not observed in the behavioral measures of task performance such as reaction time and accuracy, suggesting that blink-related neural responses during the target detection task are more sensitive in capturing aging-related brain function changes compared to behavioral measures alone. Together, these results suggest that BRO responses are not only present throughout the adult lifespan, but the effects can also capture brain function changes in healthy aging—thus providing a simple yet powerful avenue for evaluating brain health in aging.

## 1 Introduction

Spontaneous blinking occurs 15–20 times per minute, producing about 110 milliseconds of visual blackout each time (Tsubota et al., 1999; Volkmann et al., 1980). It is mediated by reciprocal activity of the orbicularis oculi and levator palpebrae superioris muscles in the face, creating a rapid closing and re-opening of the eyelids (Manning et al., 1983; Riggs et al., 1981). Although blinking has traditionally not been considered to be important in cognition, behavioral and neuroimaging studies are increasingly pointing to a potential link between the two: humans tend to blink when the attentional demand is low, such as at the ends of sentences when reading (Orchard and Stern, 1991) and during speaker pauses when listening to speech (Nakano and Kitazawa, 2010); adults have been shown to modulate their spontaneous blink behavior depending on environmental task demands (Hoppe et al., 2018; Oh et al., 2012a; Oh et al., 2012b); and blinking also activates key cortical regions associated with attentional switching in the brain (Nakano et al., 2013). These findings all suggest that spontaneous blinking also has important implications for cognitive processing.

Blink-related oscillations (BROs) are recently discovered neurophysiological responses associated with spontaneous blinking, and are believed to represent endogenous neural processes related to environmental monitoring and awareness as the brain evaluates new visual information that appears after eye re-opening (Bonfiglio et al., 2013, 2014; Liu et al., 2017, 2020; Liu et al., 2019b). BRO responses are distinct from the well-known blink oculomotor effects, and the BRO time domain activity is characterized by an increase in the delta-band (0.5–4 Hz) signal peaking approximately 250–300 ms after the blink (Liu et al., 2017, 2020). The associated cortical activations involve a wide network of posterior brain regions, including the dorsal and ventral visual streams engaged in sensory and perceptual processing (Hebart and Hesselmann, 2012), the hippocampus and parahippocampal gyri involved in spatial and episodic memory (Aminoff et al., 2013; Burgess et al., 2002), as well as the precuneus associated with numerous high-level cognitive processes such as episodic memory retrieval, visuospatial imagery, and self-related processing and awareness (Cavanna and Trimble, 2006; Gilboa et al., 2004; Kjaer et al., 2001; Liu et al., 2017; Liu et al., 2019b). In addition, BRO spectral effects encompass an early increase in signal power within the beta/low gamma (13–35 Hz) band, followed by a later and more prolonged reduction in the theta (4–8 Hz) and alpha (8–12 Hz) bands. These have been postulated to represent early sensory processing of visual information produced by blink events, followed by later higher-level episodic memory and information processing effects (Liu et al., 2017, 2020; Liu et al., 2019b).

To date, BRO responses have been demonstrated using both electroencephalography (EEG) and magnetoencephalography (MEG) across multiple task states, including resting (Liu et al., 2017; Liu et al., 2019a; Sattari et al., 2023), cognitive loading (Ghosh Hajra et al., 2021; Liu et al., 2019b), different sensory stimulation conditions (Liu et al., 2020), and complex task environments such as simulated flight (Page et al., 2024; Ziccardi et al., 2024). BRO effects have also been shown to be modulated by both task and environmental factors, as blinking during cognitive loading leads to reduced cortical activations compared to blinking during rest (Liu et al., 2019b). On the other hand, spontaneous blinking under different external sensory environments (e.g., ongoing visual vs. auditory inputs) results in altered temporal and spectral BRO response features that are consistent with the brain’s dynamic adaptation of blink processing in order to accommodate differential sensory requirements (Liu et al., 2020). Although prior studies had pointed to the potential usefulness of BRO responses in providing information about brain function, they all utilized small, relatively homogenous samples of healthy young participants, and little is known about how BRO responses may change in normal aging. Given that normal aging is known to have significant impact on brain structure and function (Harada et al., 2013; Oschwald et al., 2019)—and the precuneus region activated by BRO responses is especially sensitive to aging-related deficits such as cortical atrophy (Fjell et al., 2014), metabolic reduction (Choi et al., 2018), and decreased perfusion (Lee et al., 2009)—it is crucial to investigate BRO responses in aging in order to better understand this phenomenon. Notably, a prior study examining BRO responses in pilots during simulated flight maneuvers had demonstrated that BRO response characteristics were sensitive in detecting the effects of age in pilot performance (Ziccardi et al., 2024), but the use of a relatively low-density 14-channel EEG system limited the ability of that study to perform any in-depth characterization of age-related trajectories of BRO responses.

The present study aimed to investigate BRO effects in normal aging using a large sample of healthy adults across a broad age range. Specifically, we used data from the publicly available Cambridge Centre for Aging and Neuroscience (Cam-CAN) repository, which contains data for 700 cognitively normal healthy adults ranging in age from 18 to 88 (Shafto et al., 2014; Taylor et al., 2017). We hypothesized that BRO responses would be present and detectable throughout the adult lifespan, and that their characteristics would reflect brain changes in healthy aging. Additionally, given that BRO responses correspond to brain activity directly, we also hypothesized that BRO-based measurements would be superior to behavioral measurements such as reaction time and accuracy in detecting brain changes in healthy aging.

## 2 Methods

### 2.1 Participants

Data for this study were obtained from the Cam-CAN repository (Shafto et al., 2014; Taylor et al., 2017). Approximately 700 healthy adults were recruited for Phase 2 of the Cam-CAN study, ranging in age from 18 to 88. Volunteer recruitment was targeted to be gender-balanced between males and females, with equal distribution of individuals per age decile. A total of 631 participants had MEG data available; of these, we report results from 457 individuals (representing 72.4% of the total) following exclusions due to left-handedness (*n* = 63), noisy or corrupt MEG or electrooculogram (EOG) data (*n* = 18), missing structural MRI (*n* = 14), lack of demographic metrics such as vision test results (*n* = 18), and other exclusions related to blink behavior to be further detailed in Methods. Additional demographic and behavioral measures were also provided in the Cam-CAN data, including age, gender, number of years of continuous education, Mini-Mental State Exam (MMSE) score (Cummings et al., 2002), visual acuity using a modified Snellen eye test (Shafto et al., 2014), as well as reaction time and accuracy of task performance.

Participants were divided into four groups by age, comprising the youngest (YG, age 18−30), middle-young (MY, age 31−50), middle-old (MO, age 51−70), and oldest (OL, age 71−90) (Table 1). Demographic measures were compared across groups using one-way ANOVA, with Bonferroni correction for post-hoc multiple comparisons.

**Table 1:** Participant demographic information, presented as mean ± SD for each group.

	YG	MY	MO	OL
Group	Youngest	Middle-Young	Middle-Old	Oldest
Age	18–30	31–50	51–70	71–90
*n*	56 (28 F)	153 (75 F)	145 (81 F)	103 (51 F)
Education	16.41 ± 2.93	16.78 ± 3.15	14.86 ± 4.91^b^	13.2 ± 3.67^a^
MMSE	29.32 ± 1.16	29.16 ± 1.16	28.89 ± 1.24	28.18 ± 1.41^a^
Snellen Eye Test	0.61 ± 0.21	0.65 ± 0.20	0.74 ± 0.22^a^	0.83 ± 0.24^a^

^ap < 0.01 compared to all other groups; bp < 0.01 compared to MY.^

### 2.2 Experimental paradigm

The experimental paradigm consisted of a sensorimotor target detection task, which has been described elsewhere (Shafto et al., 2014). Briefly, participants viewed a screen with a central fixation cross, while short auditory and visual stimuli were presented either bimodally (93.8% of trials) or unimodally (6.2% of trials). Auditory stimuli consisted of 300-ms binaural tones at one of three frequencies (300, 600, or 1,200 Hz), while visual stimuli comprised bilateral checkerboards that appeared for 34 ms on either side of the fixation cross. The participants pressed a button with their right index finger whenever they detected the appearance of a target. The inter-stimulus interval ranged from 2 s to 26 s, and the duration of the overall task was 8 min 40 s.

### 2.3 Data acquisition

Data acquisition parameters for the Cam-CAN repository have also been detailed elsewhere (Taylor et al., 2017). Briefly, MEG data acquisition utilized a 306-channel Vectorview system (Elekta Neuromag, Helsinki, Finland), with participants in a seated position. Data were sampled at 1000 Hz with a bandpass filter of 0.03−330 Hz. Anatomical landmarks including the nasion, inion, and bilateral pre-auricular points were digitized to allow for co-registration between the MEG and MRI coordinate systems, and head position was also continuously monitored to allow for offline correction of head motion. Concomitant recordings of vertical and horizontal electrooculogram (vEOG and hEOG) were made, along with electrocardiogram (ECG) and task response times. High-resolution structural MRI was collected using a T1-weighted magnetization prepared rapid gradient echo (MPRAGE) sequence on a 3 T Siemens TIM Trio system with a 32-channel head coil, with 1 mm isotropic voxels.

### 2.4 Blink identification and behavioral assessments

Raw, continuous vEOG data were down-sampled to 250 Hz and visually inspected to remove artifactual channels, and blink identification was performed using a semi-automated, template matching procedure in line with prior works (Liu et al., 2017, 2020; Liu et al., 2019b). The vEOG signal was first bandpass-filtered at 0.1−20 Hz, and one blink instance that best represented a stereotypical blink was manually selected as template. This template was then convolved with the entire vEOG signal, and amplitude thresholding was applied to identify potential blink instances. In order to minimize contamination from adjacent blink events, temporal thresholding was also applied to quantify the time interval between adjacent blinks, then exclude any blink events that were <3 s apart. The total number of blinks was quantified prior to temporal thresholding to enable behavioral evaluation of blink rate. To ensure a sufficient number of blink trials for BRO extraction, only participants with more than three trials following temporal thresholding were included in further analysis, in accordance with prior studies (Liu et al., 2020; Liu et al., 2019b). A total of 63 participants were excluded due to frequent blinking, as insufficient number of blink trials remained following temporal thresholding.

**Figure 1:**
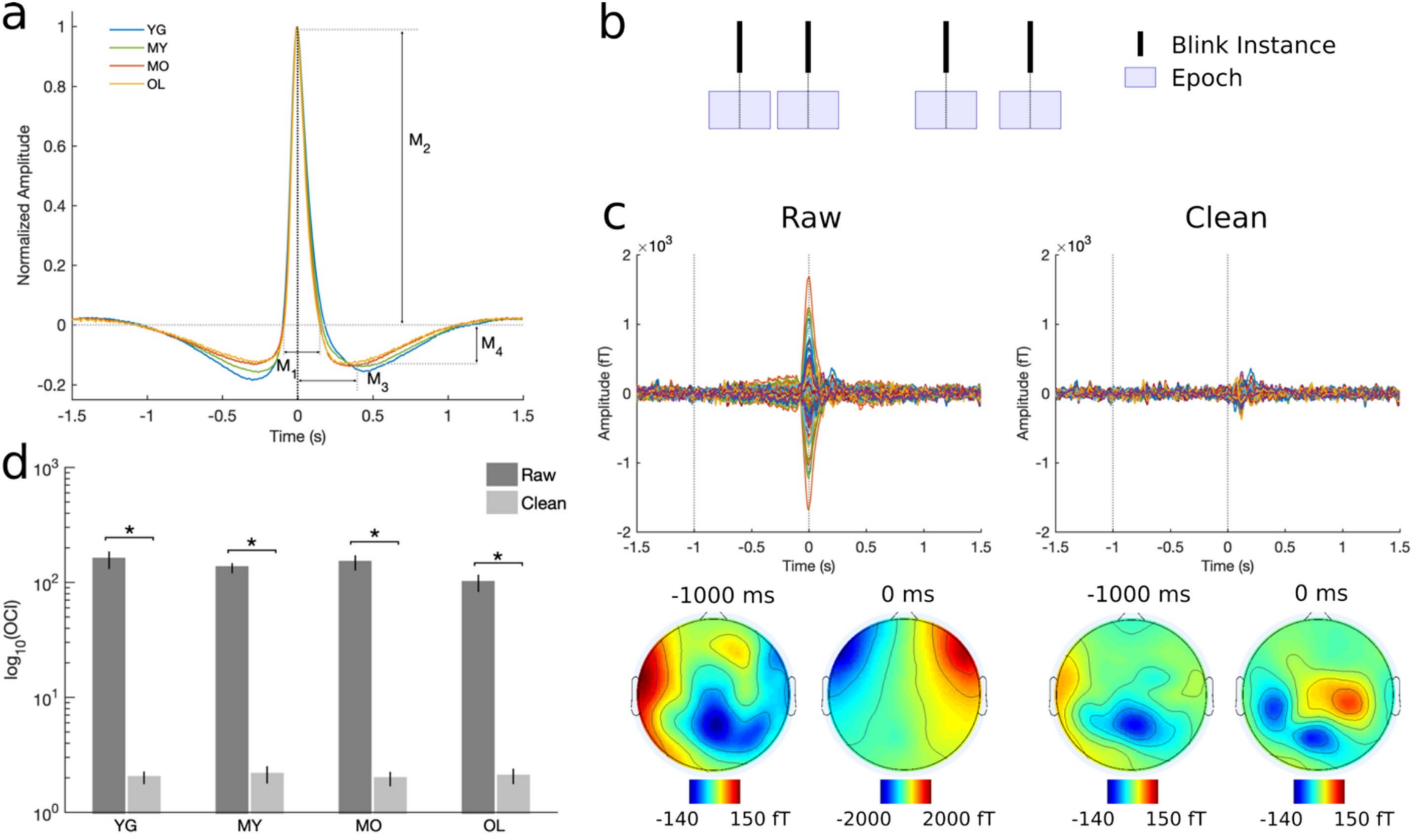
Preprocessing and artifact removal results. **(A)** Grand-averaged normalized vEOG blink traces showing the quantitative morphological features used to assess blink behavioral characteristics. M_1_ = positive peak width; M_2_ = positive peak amplitude; M_3_ = negative peak time; M_4_ = negative peak amplitude. Black dotted line at 0 ms denotes the moment of complete eye closure or T_0_. **(B)** Schematic illustration of data segmentation technique. BRO effects were assessed by segmenting data into 3-s epochs centered at 0 ms latency or T_0_. **(C)** Representative subject MEG data before and after artifact removal. Top panel shows all-channel BRO waveforms after averaging across all blink trials, while bottom panel shows corresponding scalp topographies. Dotted lines denote the latencies of maximum blink amplitude (0 ms) and pre-blink baseline (−1000 ms). **(D)** Ocular contamination index (OCI) results show 98% reduction in blink signal contribution after artifact removal. *p < 0.0001.

Blink behavior was assessed via both qualitative and quantitative methods in line with previous literature (Liu et al., 2017, 2020; Liu et al., 2019b). For qualitative assessment, individual-level trial-averaged vEOG waveforms were normalized by their respective maximum amplitudes before averaging across subjects in each group to minimize bias due to differential voltages in the raw blink signal. For quantitative measures, morphological features were extracted corresponding to the height and width of different regions in the un-normalized individual-level blink waveforms (Figure 1A). The Cam-CAN dataset also provided task performance measurements in the form of reaction time and target detection accuracy, and these were compared across age groups using one-way ANOVA with Bonferroni correction for post-hoc multiple comparisons.

### 2.5 MEG preprocessing

We utilized the preprocessed MEG data from the Cam-CAN repository, in which temporal signal space separation (tSSS) had been applied to remove noise from both external sources and head position indicator coils, head motion was corrected, noisy channels reconstructed, and each dataset had been transformed to a common head position (Taulu et al., 2005; Taylor et al., 2017). Subsequent analyses utilized a combination of SPM12 (Henson et al., 2019), EEGLAB (Delorme and Makeig, 2004), and custom scripts in MATLAB (The Mathworks Inc.).

Continuous MEG data were down-sampled to 250 Hz and visually inspected to remove artifactual channels. Data were then notch-filtered at 50 Hz and 100 Hz to remove mains power (5 Hz bandwidth), and bandpass-filtered at 0.1−80 Hz using a zero-phase 4th-order butterworth filter. Independent component analysis (ICA) was performed using the InfoMax algorithm to identify and remove contamination due to environmental and physiological sources such as blinks, saccades, cardiac activity, muscle contraction, breathing, and movement based on their stereotypical characteristics (Delorme and Makeig, 2004; Ghosh Hajra et al., 2018; Hajra et al., 2020; Liu et al., 2019b; Makeig et al., 1996). For instance, the blink artifact involves a large positive spike in the time-domain event-related field signal occurring at blink latency, with the largest signals concentrated near the frontal eye regions. Following artifact removal, the cleaned continuous MEG data were subsequently segmented into 3-s epochs centered on the latency of maximum blink amplitude, or *T_0_*, to enable BRO extraction (Figure 1B).

### 2.6 Effectiveness of artifact removal

To ensure complete removal of artifact prior to BRO extraction, the effectiveness of the ICA-based artifact removal procedure was rigorously assessed using both quantitative and qualitative techniques (Liu et al., 2017, 2020; Liu et al., 2019b). Qualitative evaluation involved visual inspection of the trial-averaged, individual-level data before and after artifact removal to ensure elimination of temporal and topographical features corresponding to ocular artifact. Quantitative assessment was performed by computing the ocular contamination index (OCI) for each dataset as the ratio of ocular signal contribution at the maximum blink latency (0 ms) relative to pre-blink baseline (−1000 ms) in accordance with previously published methods (Liu et al., 2020). Statistical comparisons were made using two-tailed, paired *t*-test between the raw and cleaned data.

### 2.7 Global field power

**Figure 2:**
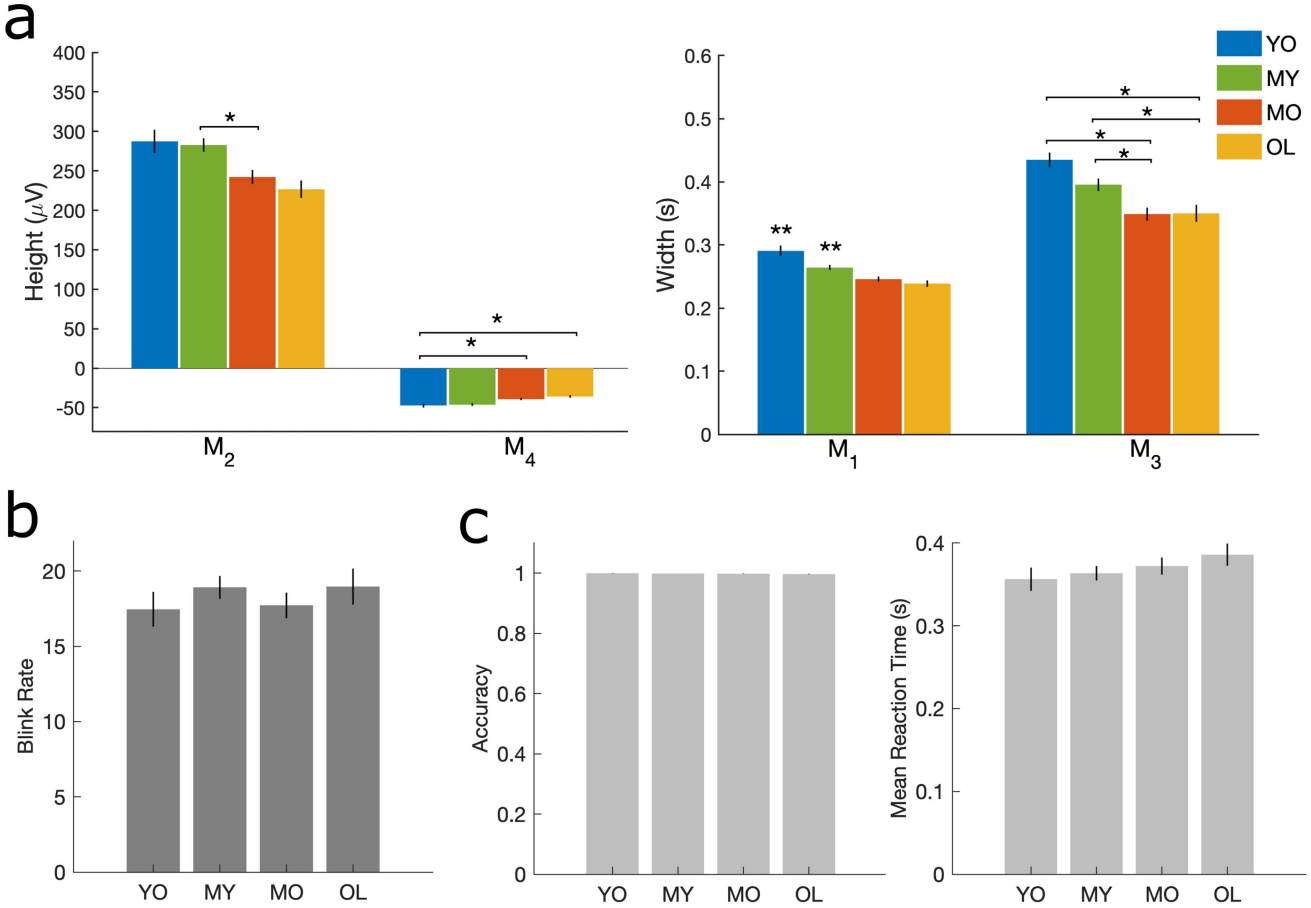
Measures corresponding to blink morphology and task performance. **(A)** Height and width of vEOG waveform. M_2_ = positive peak amplitude; M_4_ = negative peak amplitude; M_1_ = positive peak width; M_3_ = negative peak time. **(B)** Number of blinks per minute. **(C)** Target detection task performance, including accuracy (left) and mean reaction time (right). All results are computed at the individual level and presented as mean ± SE across participants. *p < 0.05 as indicated. **p < 0.05 compared to all other groups.

Time-domain BRO effects at the sensor level were measured using global field power (GFP) to quantify the spatial variance across channels (Liu et al., 2017; Skrandies, 1990). Cleaned continuous data were bandpass-filtered into the delta band (0.5−4 Hz), segmented into 3-s blink epochs, averaged across trials, and GFP was derived for each participant before grand-averaging across subjects. Windows of interest were identified corresponding to salient features in the grand-averaged waveform (Figure 2A), including the two post-blink peaks (P1, 50−180 ms and P2, 220−350 ms), a pre-blink baseline (B1, -1300 to -1100 ms), and a blink preparation interval just before blink onset (B2, -350 to -150 ms). Mean GFP magnitudes were computed within these windows of interest for each individual. The latencies of the P1 and P2 peaks were measured for each participant and averaged across subjects for each group. For individuals with only one post-blink GFP peak, only P2 latency was determined based on the grand-averaged GFP waveform, while P1 was excluded from group averaging. Quantitative measures were statistically compared across groups using one-way ANOVA with Bonferroni correction.

### 2.8 Source localization

Source localization was performed using whole-brain analysis in SPM12 according to previously published procedures (Liu et al., 2017; Liu et al., 2019b). Following standard forward modeling using a single-shell spherical head model, source reconstruction was performed using minimum norm estimates with group constraints during inversion to improve source reliability across subjects (Hauk, 2004; Litvak and Friston, 2008). Source reconstruction used denoised, segmented, and trial-averaged data (0.5–80 Hz) over the entire 3-s epoch, and time-frequency contrast images were generated by averaging the estimated source activity over the delta frequency band and across the previously selected windows of interest corresponding to the two post-blink GFP peaks (P1, P2) and the pre-blink baseline (−1300 to −1100 ms). These contrast images were projected to a 3-dimensional source space, smoothed using a Gaussian kernel with 8 mm full-width at half-maximum, and entered into a mixed-effects general linear model (GLM) using two-way ANOVA, with *time* (i.e., B1 vs. P1, B1 vs. P2, B1 vs. B2) as a within-subject factor and *age group* as a between-subject factor. Individual participant age and visual acuity were incorporated as covariates in the model to account for inter-individual differences in participant vision and age.

### 2.9 Source-level spectral effects

Source-level timeseries were extracted using virtual electrodes positioned at coordinates centered within activation clusters located in the bilateral precuneus [MNI coordinates (8 −72 38), (−8 −76 46)]. Voxel time courses were smoothed over a spherical volume of interest with 5-mm radius, filtered to 0.5–45 Hz, and event-related spectral perturbation (ERSP) was computed using continuous wavelet transform (CWT) with the Morlet function and 6 cycles (Liu et al., 2017; Liu et al., 2019b; Makeig, 1993). CWT was performed for each trial and virtual electrode, and the log power was computed as the logarithm of the squared absolute values of the wavelet coefficients. Baseline correction was performed by subtracting from each trial the mean log power of the pre-blink baseline window defined as -1500 to -500 ms in line with prior works (Liu et al., 2017, 2020). Results were then trial-averaged for each individual and grand-averaged in each group.

Blink-related effects were statistically assessed using a nonparametric permutation approach based on Monte Carlo estimates (Liu et al., 2020; Maris and Oostenveld, 2007). A group-level paired t-test was first computed between the trial-averaged log spectral power in the pre-blink and post-blink intervals at each frequency and time point, with *p* < 0.05 for two-tailed significance threshold. The data were then randomly permuted between the two intervals for each frequency, and new T-statistic values were derived. This was repeated 1,000 times, and a distribution of permuted *T*-values was generated. The true T statistic between the pre-blink and post-blink intervals was then compared to the permuted distribution to determine probabilities, and results were deemed significant if *p* < 0.05. This process was carried out separately for each age group to assess statistically significant blink-related spectral features.

Quantitative comparisons of spectral features in each frequency band were made by selecting pre- and post-blink intervals corresponding to known BRO effects, including those in the pre-blink interval [i.e., (−1000 −600), (−600 −300), and (−300 0) ms], and the post-blink interval [i.e., (0–300), (300–1000) ms]. Mean spectral values were calculated for each individual within each time window and frequency, and grand-averaged across participants. Statistical assessment was conducted using one-way ANOVA with *age* as a between-subject factor, with Bonferroni correction for post-hoc multiple comparisons.

## 3 Results

### 3.1 Artifact removal

Qualitative examination of BRO signals before and after artifact removal revealed that features such as a large signal spike at blink latency (0 ms or *T_0_*) and frontally concentrated topography that are consistent with ocular artifact were eliminated after denoising (Figure 1C). Additional quantitative results using OCI showed that the maximal blink signal contribution was reduced by more than 98% following artifact removal (Figure 1D). This is consistent with prior BRO literature (Liu et al., 2017, 2020), and indicates that these procedures effectively removed ocular contamination due to blinking.

### 3.2 Behavior

Behavioral assessments showed that blink morphology exhibited age-related reductions in both the height (M_2_, M_4_) and width (M_1_, M_3_) of the vEOG waveform (Figure 2A). These features correspond approximately to the speed (M_1_) and amplitude (M_2_) of the blink itself, as well as the speed (M_3_) and amplitude (M_4_) of post-blink recovery (Iwasaki et al., 2005). Such reductions in blink kinematics are consistent with aging-related blepharoptosis or droopiness of the eyelid, which reduces the palpebral fissure width and limits the amplitude and peak velocity of blink-induced eyelid closure in older adults (Sun et al., 1997). However, no age effects were found in blink rate or task behavioral measures of mean reaction time and accuracy (Figures 2B,C), consistent with prior studies (Gruber et al., 2014; Sforza et al., 2008). These results indicate that normal aging did not alter the rate of blinking or performance in the target detection task, and the observed blink kinematic differences are likely the passive consequences of aging-related weakening in the eyelid muscles (Sun et al., 1997).

### 3.3 Sensor-level time-domain effects

**Figure 3:**
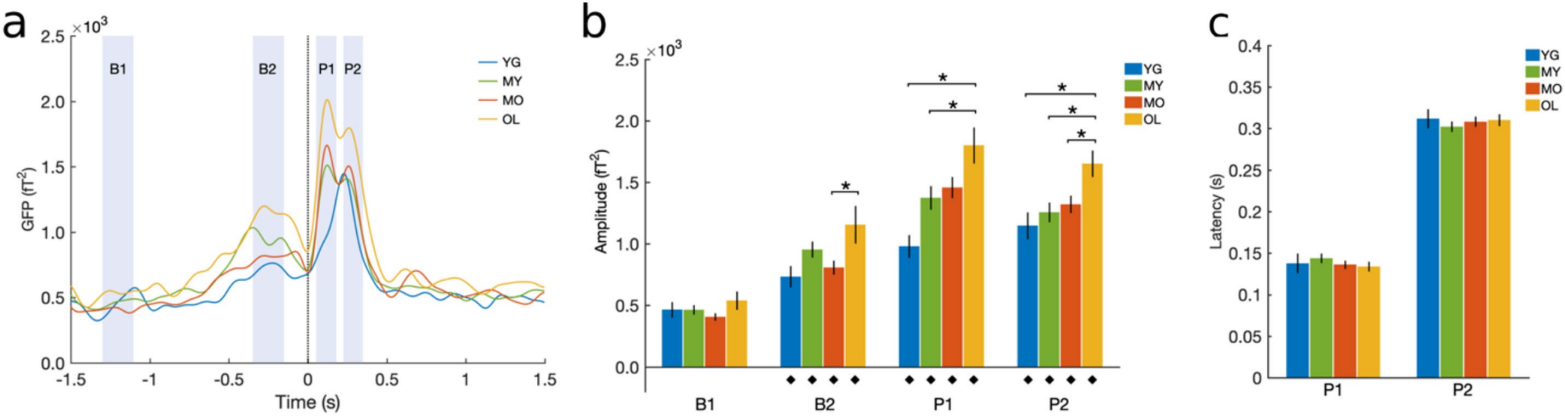
Sensor-level GFP results. **(A)** Grand-averaged GFP waveform. Black dotted line denotes latency of blink maximum or T_0_. Shaded regions denote windows of interest spanning different features of the BRO waveform, including post-blink BRO peaks (P1 and P2), blink preparation interval (B2), and pre-blink baseline (B1). **(B)** Mean GFP amplitude within the specified intervals of interest. **(C)** Latency of the post-blink peaks. Results computed for each participant and presented as mean ± SE across subjects. ^♦^*p* < 0.0001 compared to B1; *p < 0.05 as indicated.

GFP analysis was performed to examine BRO temporal effects at the sensor level. Results showed that BRO responses were present in all age groups, with morphological features consistent with prior literature (Bonfiglio et al., 2013; Liu et al., 2017, 2020; Liu et al., 2019a; Liu et al., 2019b). Three of the four groups exhibited a bifurcated morphology with two post-blink peaks P1 and P2 maximal at approximately 130 ms and 320 ms, respectively, while the youngest (YG) group exhibited a single peak at a comparable latency to that of P2 (Figure 3A). GFP amplitudes for P1, P2, and B2 were all increased compared to the B1 in each group (*p* < 0.0001), indicating that the BRO effects were significantly different from baseline for all ages (Figure 3B). In addition, all peaks showed age-related increase in GFP amplitude except B1, indicating that BRO sensor activity increased with age during both the post-blink peaks (P1 and P2) as well as the blink preparation interval (B2), and these effects were not due to potential age differences in baseline brain activity (B1, Figure 3B). Peak latency did not differ across groups for either of the post-blink peaks, indicating that the speed of BRO processing did not change with age (Figure 3C).

### 3.4 Source-level effects

**Figure 4:**
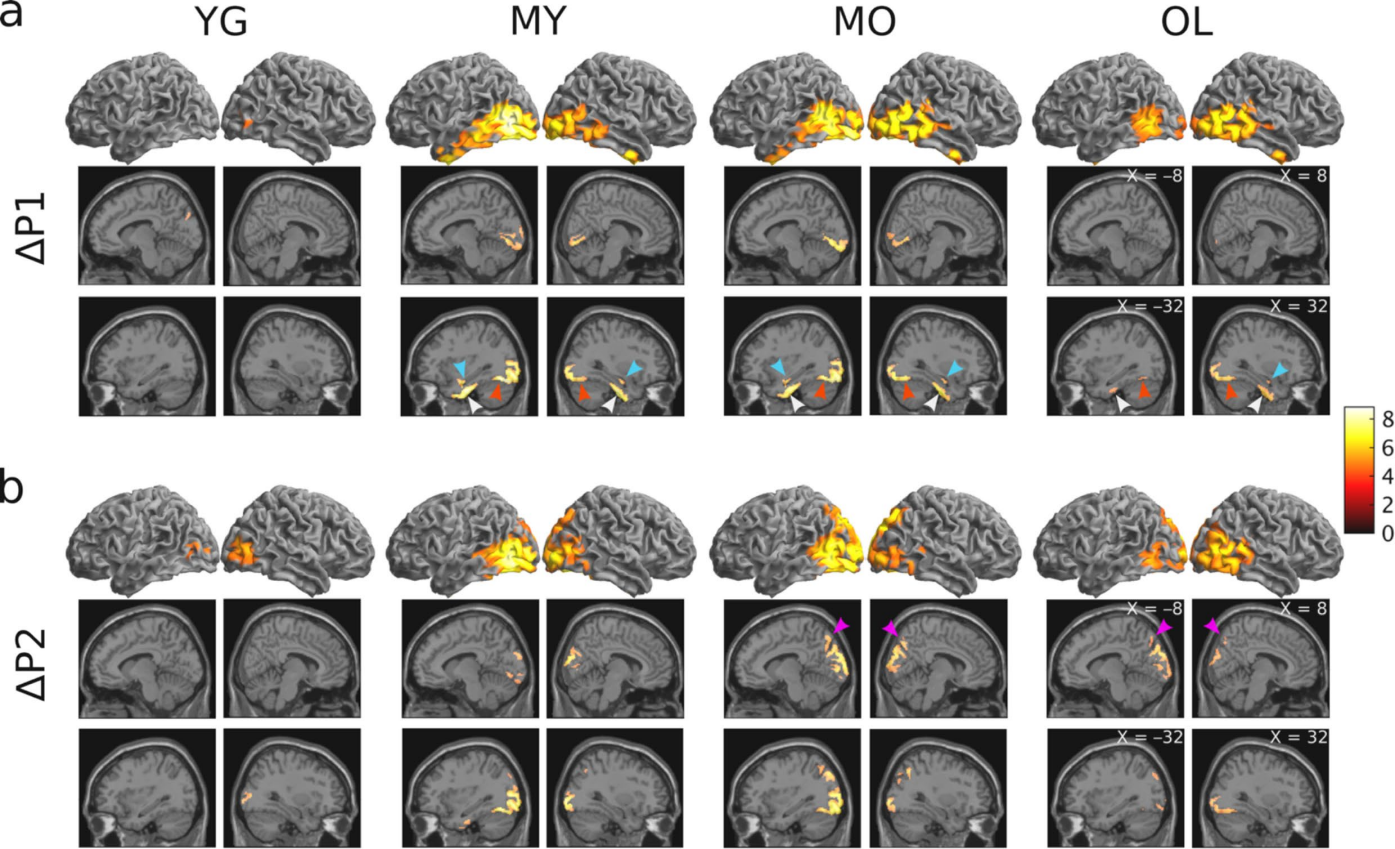
Results of whole-brain source localization analysis showing blink-related increase in brain activation for each age group (*p* < 0.05 FWE). **(A)** Blink-related cortical activations during the P1 peak compared to baseline (ΔP1 = P1 – B1 contrast). **(B)** Blink-related cortical activations during the P2 peak compared to baseline (ΔP2 = P2 – B1 contrast). Color bar denotes T-statistic values. Cyan arrows = hippocampus; white arrows = parahippocampal gyri; red arrows = fusiform; magenta arrows = precuneus.

**Figure 5:**
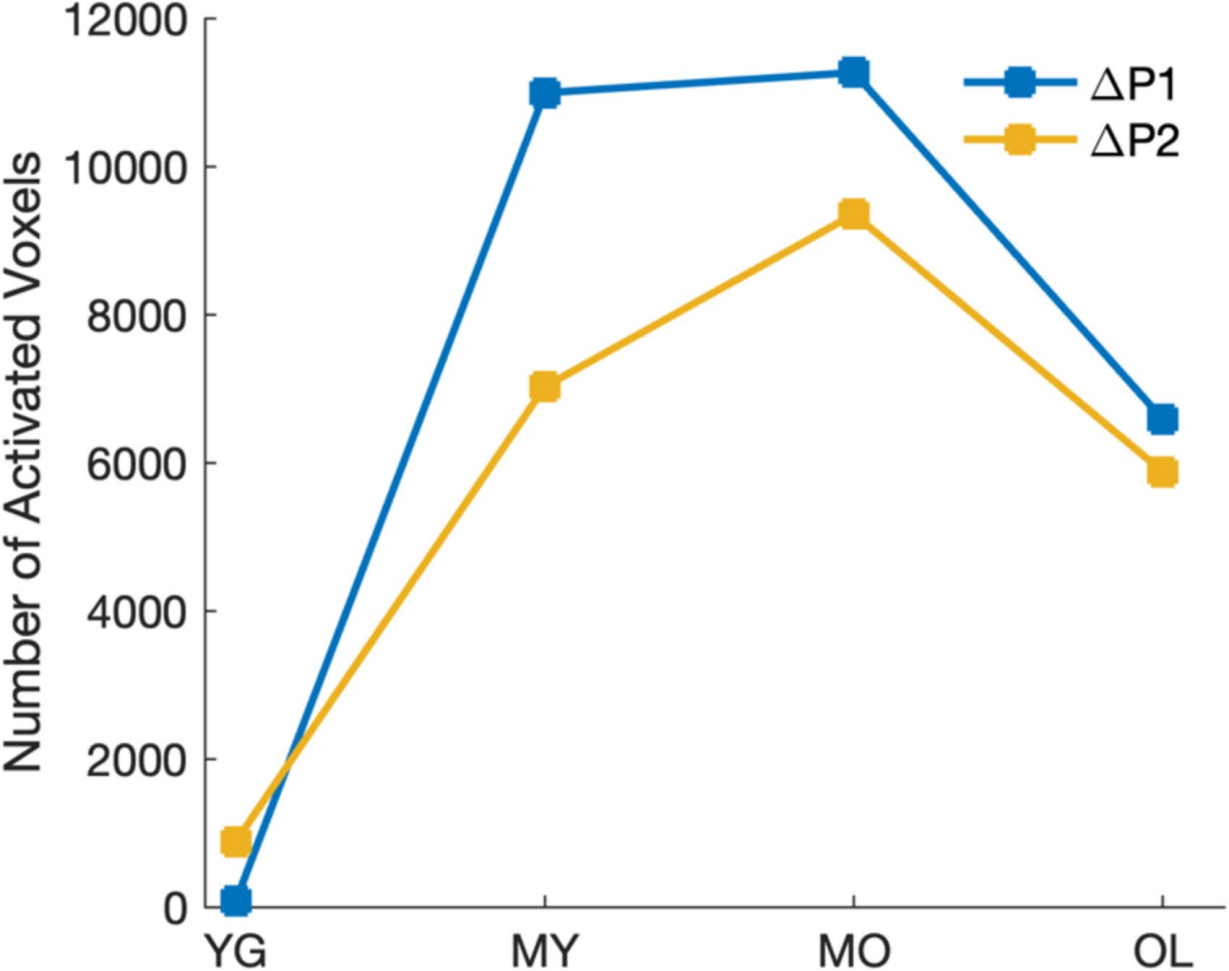
Extent of cortical activations in each post-blink peak showing inverted-U relationship with age.

To determine blink-related cortical activations and the impact of aging, source localization was performed for all age groups. Results showed that BRO responses were present in all age groups, and the blink-related activations are in line with prior literature (Liu et al., 2017; Liu et al., 2019b). Compared to pre-blink baseline, BRO responses during the first window post-blink (ΔP1, 50–180 ms) led to increased activity in the bilateral occipital and posterior temporal regions along with the hippocampus, parahippocampal gyri, and fusiform (Figure 4A), while the second window (ΔP2, 220–350 ms) showed increased activity in the bilateral occipital, temporal, and posterior parietal regions along with the precuneus (Figure 4B). Minimal BRO-related activations were observed in the YG group, with only small occipital clusters in P1 which expanded in P2. The extent of BRO-induced cortical activations exhibited an inverted-U relationship with age, with the total number of activated voxels peaking in MO (Figure 5). No significant effect of age or visual acuity was found, indicating that the observed effects were not due to individual variations in age or vision. Additional comparisons examining blink-related activations during the blink preparation interval (i.e., B2–B1 contrast) did not produce any suprathreshold clusters, suggesting that blink preparatory neural processes did not lead to cortical activations significantly different from baseline, despite the observed sensor-level differences.

**Figure 6:**
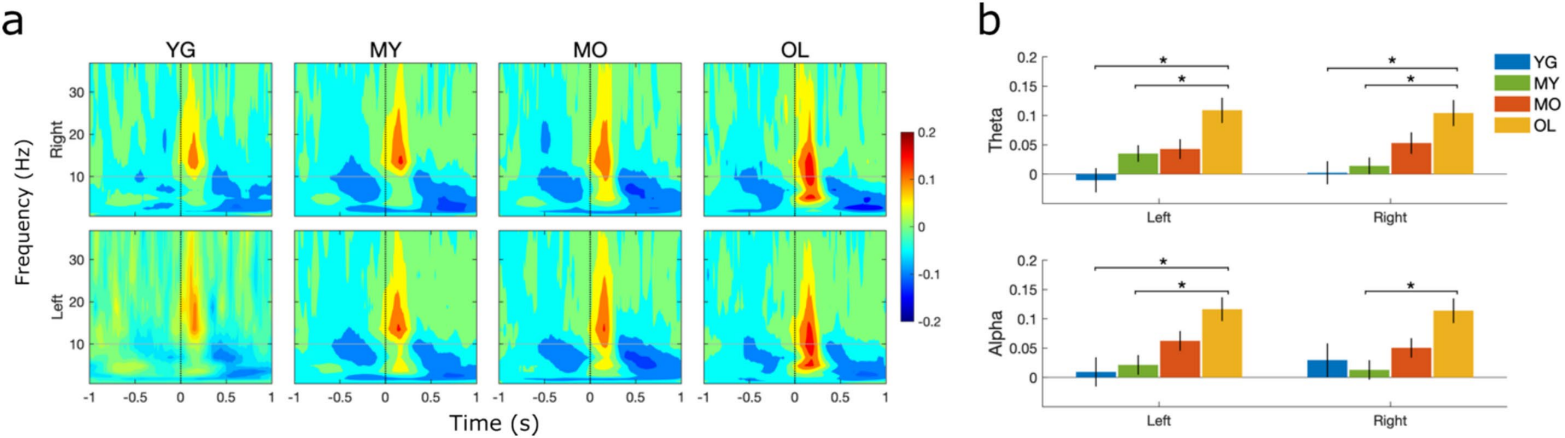
Source-level spectral effects within the left and right precuneus. **(A)** Grand-averaged log spectral power. Black dotted line denotes T_0_ or latency of blink maximum. Color bar denotes log power values. **(B)** Mean spectral power within the theta and alpha bands during the 0–300 ms post-blink interval. Values were computed at the individual level and presented as mean ± SE across participants. *p < 0.05.

Time-frequency analysis was undertaken to examine source-level BRO spectral effects within the bilateral precuneus. Results revealed an early blink-related increase in signal power, or event-related synchronization (ERS), in the beta/low gamma bands (13–35 Hz) during the 0–300 ms interval post blink. This is followed by a later and more prolonged power reduction, or event-related desynchronization (ERD), in the theta (4–8 Hz) and alpha (8–12 Hz) bands during the 400–1000 ms interval (Figure 6A). There was also a pre-blink ERD in the theta/alpha bands during the −600 to 0 ms interval before blink onset, with low-magnitude extension into the higher frequency bands. These effects were observed in all age groups and consistent with prior literature (Liu et al., 2020; Liu et al., 2019b), suggesting that BRO effects were present within the bilateral precuneus throughout the lifespan.

To evaluate age differences in spectral effects within the bilateral precuneus, the mean spectral power in different frequency bands and time windows of interest were compared using one-way ANOVA. Results showed significant age-related increase in BRO signal power within the theta and alpha bands during the early post-blink interval (0–300 ms) in both the left and right precuneus (Figure 6B), while no age-related differences were observed in other intervals or frequency bands. These results suggest that the early post-blink theta- and alpha-band BRO effects are sensitive to brain changes in normal aging. Additionally, to ensure that the observed effects were not due to the particular grouping of participants by age in this study, further analyses were conducted to compare the precuneus spectral effects by dividing participants into both 10-year (Supplementary Figure S1) and 5-year age groupings (Supplementary Figure S2). The same age-related increase was found in both the other groupings.

## 4 Discussion

In this study, we conducted the first investigation of BRO responses in healthy aging using a large, cross-sectional sample of cognitively normal healthy adults across a wide age range (ages 18–88). Our results demonstrate the presence of BRO responses throughout the adult lifespan, and also show age-related modulations in BRO effects that suggest these blink-related neural responses can capture brain changes in healthy aging.

### 4.1 BRO responses are present across the adult lifespan

We first examined BRO effects at the sensor level using GFP to capture time-domain activity across all sensors. Results showed that GFP activity exhibited increased amplitude during two post-blink intervals compared to the pre-blink baseline, and the effects were observed in all age groups (Figure 3). This is consistent with prior literature (Liu et al., 2017, 2020; Liu et al., 2019a; Liu et al., 2019b), and confirms the presence of BRO responses across all age groups.

To determine the neuroanatomical origins of the BRO response, we performed source localization to assess cortical activations underlying the two post-blink peaks in GFP. Our results showed that BRO effects engaged brain regions including: (1) the bilateral occipital, posterior parietal, and inferior temporal regions consistent with the dorsal and ventral visual processing pathways (Hebart and Hesselmann, 2012); (2) the bilateral precuneus known to be associated with many aspects of high-level cognition including visuospatial processing, episodic memory retrieval, and self-related processing (Cavanna and Trimble, 2006; Gilboa et al., 2004; Kjaer et al., 2002; Wenderoth et al., 2005); (3) the hippocampus and parahippocampal gyri corresponding to spatial and non-spatial episodic memory and association processing (Aminoff et al., 2013; Burgess et al., 2002); (4) the fusiform gyri associated with complex image encoding and face perception (Machielsen et al., 2000; Weiner and Zilles, 2016); and (5) the bilateral anterior temporal lobes (ATL) associated with semantic cognition and the mental representation of meaning (Visser et al., 2010) (Figure 4). These effects are consistent with prior literature (Bonfiglio et al., 2013; Liu et al., 2017; Liu et al., 2019b), and demonstrate the presence of the BRO response throughout the adult lifespan.

At the source level, our analysis focused on spectral effects within the bilateral precuneus as these regions are known to be involved in BRO processing (Liu et al., 2017; Liu et al., 2019b). Our results showed that BRO responses exhibited beta/low gamma (13–35 Hz) band ERS during the early time window immediately after the blink (0–300 ms), followed by theta (4–8 Hz) and alpha (8–12 Hz) ERD during the later interval (300–1000 ms). These effects are also in line with prior studies (Liu et al., 2017, 2020; Liu et al., 2019a; Liu et al., 2019b), and confirm the presence of BRO responses within the precuneus in all age groups (Figure 6). Together, these results demonstrate that BRO responses are present throughout the adult lifespan, and the associated effects are detectable across the sensor and source levels.

### 4.2 BRO response amplitudes increase with healthy aging

We assessed age-related effects in BRO processing by examining sensor-level GFP as well as source-level activity within the bilateral precuneus as these regions are highly implicated in aging-related brain changes such as cortical atrophy and metabolic reduction (Fjell et al., 2009; Kalpouzos et al., 2009). Our results showed that both GFP amplitudes and BRO spectral power within the precuneus exhibited age-related increase (Figures 3, 6), while GFP peak latencies were not different with age. These results suggest that BRO responses are sensitive to brain function changes in healthy aging, but these changes did not alter the speed of information processing following blinking.

In the spectral domain, age-related increases were found in BRO theta and alpha ERS during the early post-blink interval (0–300 ms, Figure 6B). Blink-related theta and alpha oscillations have been postulated to represent episodic memory and information processing effects, respectively (Greenberg et al., 2015; Klimesch, 2012; Liu et al., 2019b; Matsumoto et al., 2013). In particular, alpha ERD is believed to index reduced cortical inhibition or increased neuronal excitability associated with active information processing (Kelly et al., 2006; Klimesch, 2012; Sauseng et al., 2009), while precuneus theta ERD has been shown to be crucial in facilitating associative episodic memory (Greenberg et al., 2015). These findings are in line with the postulated role of precuneus theta and alpha oscillations in BRO-related episodic memory and information processing effects. Given that the theta and alpha ERD both occur during the late post-blink interval (300–1000 ms), the preceding theta and alpha ERS during the early interval (0–300 ms) may indicate neural preparatory efforts in anticipation of upcoming blink processing. As such, the observed age-related increase in the early theta and alpha ERS may correspond to greater preparatory efforts being needed as a result of decreased neural efficiency with aging (Buckner, 2004; Gold et al., 2013; Mather and Harley, 2016; Nyberg et al., 2009, 2012; Vogel et al., 2005), which has been shown to compromise cognitive performance (Colcombe et al., 2005) and may thus impair the ability of the brain to process blink-related information. Such reduction in neural efficiency may also reflect underlying aging-related neurodegeneration such as cortical thinning, which is known to be prominent in the precuneus (Fjell et al., 2014; Storsve et al., 2014). Interestingly, the late theta and alpha ERD itself does not change with age (Supplementary Figure S5), suggesting that the blink-related neural processing remains stable with healthy aging.

No significant age-related differences were found in the pre-blink spectral effects. However, the observed pre-blink beta ERD is consistent with a previous study examining BRO effects during ongoing visual stimulation (Liu et al., 2020), in which beta ERD was postulated to represent the suppression of ongoing visual processes in preparation for processing the upcoming blink. The visual stimulus presentation employed in the target detection task in the current study likely also resulted in similar suppression of ongoing visual processes prior to blink onset. In a similar manner, the pre-blink alpha ERD in our study likely also reflects suppression of inhibition prior to blink onset, in preparation for processing the upcoming blink (Klimesch, 2012). The pre-blink theta ERD in our study may be related to expectation of upcoming sensory input, as a prior study also found similar effects preceding the onset of pain stimulus when individuals were expecting the input (Taesler and Rose, 2016). The absence of significant age differences in pre-blink effects suggests that these pre-blink BRO processes remain stable with healthy aging.

### 4.3 BRO cortical activations exhibit inverted-U relationship with age

We observed an inverted-U relationship with age in BRO cortical activations, such that the activation extent in both P1 and P2 intervals increases with age from the youngest group, peaks in the MO group (age 51–70), then decreases again in the oldest group (Figure 5). This suggests greater cortical recruitment for BRO processing with age, which is likely due to decreased neural efficiency with aging (Gold et al., 2013). These observed effects are consistent with the Scaffolding Theory of Aging and Cognition (STAC), in which the brain recruits additional neural resources as a compensatory mechanism in order to maintain cognitive performance when faced with age-related brain degeneration (Oschwald et al., 2019; Park and Reuter-Lorenz, 2009; Stern, 2002). As gray matter volume is known to decrease linearly with age beginning around the age of 20 (Fjell et al., 2013; Giorgio et al., 2010), this may help to account for the gradual expansion of BRO cortical activations with age. However, as the amount of functional loss that can be mitigated through neural compensation is also constrained by the total neural resources available at any given time (Oschwald et al., 2019; Park and Reuter-Lorenz, 2009; Stern, 2002), the accumulation of age-related neurodegeneration over time in the oldest group likely reduced the overall neural resources available in that group compared to MO, leading to decreased activations. This is also in line with the cognitive performance results from MMSE, in which OL showed lower scores compared to all other groups (Table 1). Additionally, the observed inverted-U relationship with age in cortical recruitment is also consistent with prior studies demonstrating a similar pattern of aging-related functional and structural changes in the brain, including episodic memory capacity (Rönnlund et al., 2005), cerebral white matter volume (Fjell et al., 2013), as well as hippocampal volume (Fjell et al., 2013). Although this inverted-U relationship is different than the age-related increase seen in the GFP and spectral effects, we postulate that the age-related increase may be due to the increased cortical “effort” in BRO processing as a result of decreased neural efficiency with aging (Colcombe et al., 2005), while the inverted-U relationship with age may reflect contributions from both decreased neural efficiency as well as functional neurocompensation in aging (Oschwald et al., 2019). The observed patterns of BRO changes with age in our study thus encompass both the neural “effort” in processing information, as well as the availability of neural resources which constrains the cumulative cortical effect. Nevertheless, further studies are needed to better elucidate the precise mechanisms of age-related changes in BRO processing.

Besides the age-related expansion of BRO cortical activations, there is also a staggered recruitment of cortical regions across the different age groups, in that the hippocampal and parahippocampal activation begins in MY (age 31–50) and remains present up to the oldest group, while the precuneus activation begins in MO (age 51–70) and remains present up to the oldest group (Figure 4). This suggests a potential systematic expansion of the neural recruitment for BRO processing with age, such that specific neural resources are gradually brought onboard to compensate for the accumulation of neurodegeneration with aging.

It is interesting that no significant cortical activations were observed in the precuneus, hippocampus, or parahippocampal regions for the youngest group (age 18–30) in our study. This is contrary to prior studies of BRO responses which had reported significant cortical engagement in these regions (Liu et al., 2017; Liu et al., 2019b). Nonetheless, there are experimental differences between those studies and ours which may have led to the observed differences. For instance, those studies utilized passive task paradigms with either no sensory input (Liu et al., 2017; Liu et al., 2019b), or only auditory or visual inputs in isolation (Liu et al., 2020), and none had required active task performance to maintain vigilance. On the other hand, the current study utilized data collected under dynamic environmental conditions with multisensory inputs, and also required active responses to indicate detection of short-duration targets. This ensures a higher level of participant vigilance in our study, and may have altered the neural resource allocation involved in BRO processing relative to tasks with less complex sensory inputs and lower vigilance states. Interestingly, although prominent age-related effects were seen in BRO processing during this task, the behavioral measures of task performance such as reaction time and accuracy were not different across age groups (Figure 2). This suggests that BRO neural processing during the target detection task has superior sensitivity in detecting changes due to normal aging compared to the task itself.

Finally, it should be noted that the use of minimum norm estimates for source localization in this study has some limitations, in that the technique has a tendency to bias towards the cortical surface due to its regularization parameter which requires minimum source power (Litvak and Friston, 2008). However, this approach has the advantage of requiring minimal assumptions about cortical sources (Hauk, 2004), and is in line with previous BRO studies (Liu et al., 2017; Liu et al., 2019b). Nonetheless, future studies should confirm the source activation findings using alternative approaches, such as different regularization techniques for minimum norm estimates (Hincapié et al., 2016; Vallarino et al., 2023) as well as spatial filtering with beamformer (Hillebrand et al., 2005; Hu et al., 2017). Additionally, given the present study is the first investigation of aging-related changes in BRO responses and uses a single cohort of a large sample of healthy adults, the observed findings should also be validated in future studies using other cohorts of participants.

## 5 Conclusion

We conducted the first investigation of blink-related oscillations in healthy aging using a large sample of healthy adults across a wide age range (age 18–88). Our results demonstrate that BRO responses are not only present throughout the adult lifespan, but there are also substantial age-related modulations that reflect underlying neural compensation. These age effects are not present in behavioral measures of task performance such as reaction time and accuracy, indicating that BRO responses have greater sensitivity in capturing brain changes in healthy aging compared to behavior alone. These findings significantly advance our understanding of the BRO phenomenon, and demonstrate the potential of blink-related neural processing for detecting brain changes in normal aging.

## Data Availability

Publicly available datasets were analyzed in this study. This data can be found at: Cambridge Centre for Aging and Neuroscience, https://cam-can.mrc-cbu.cam.ac.uk/dataset/.

## Group members of Cam-CAN Group

Cambridge Centre for Aging and Neuroscience, University of Cambridge, Cambridge, United Kingdom.
