# Supplementary material for: Spontaneous blinking and brain health in aging: Large-scale evaluation of blink-related oscillations across the lifespan
Source: Front Aging Neurosci. 2025 Jan 7;16:1473178. doi: 10.3389/fnagi.2024.1473178 (PMC11747640; doi:10.3389/fnagi.2024.1473178)
Supplement: Supplementary file 1 [file Data_Sheet_1.pdf]

Supplementary Information

To ensure that the observed age-related increase in BRO effects was not due to the particular grouping of participants into 20-year bins, we also examined BRO effects using both 10-year and 5-year age bins. Results showed that the same age-related increase was found in both the 10-year and 5-year age groupings across both sensor and source levels.

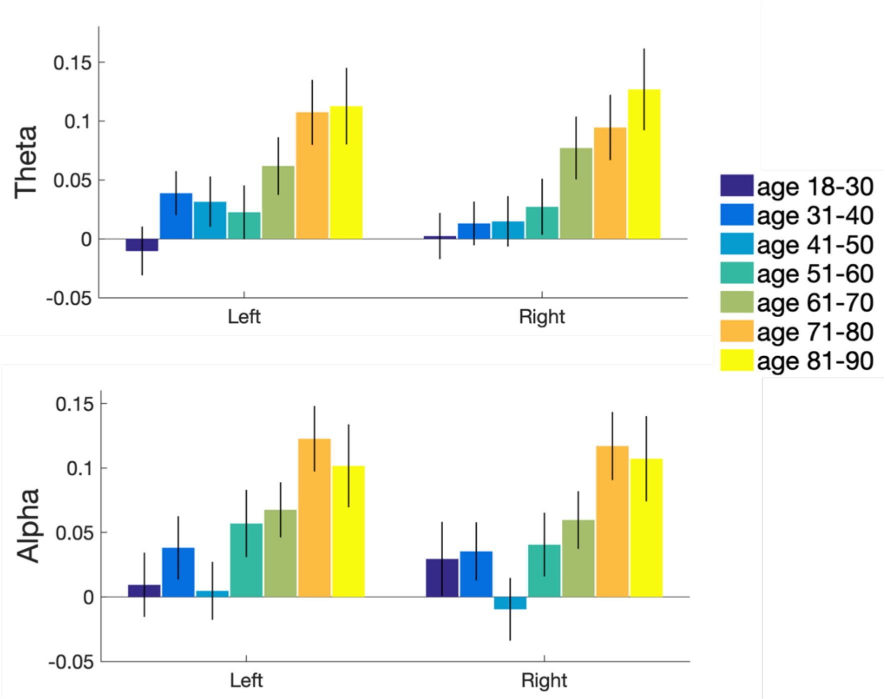

Figure S1. BRO source-level spectral effects in the theta and alpha bands within the left and right precuneus, after dividing participants into age groups by 10-year bins.

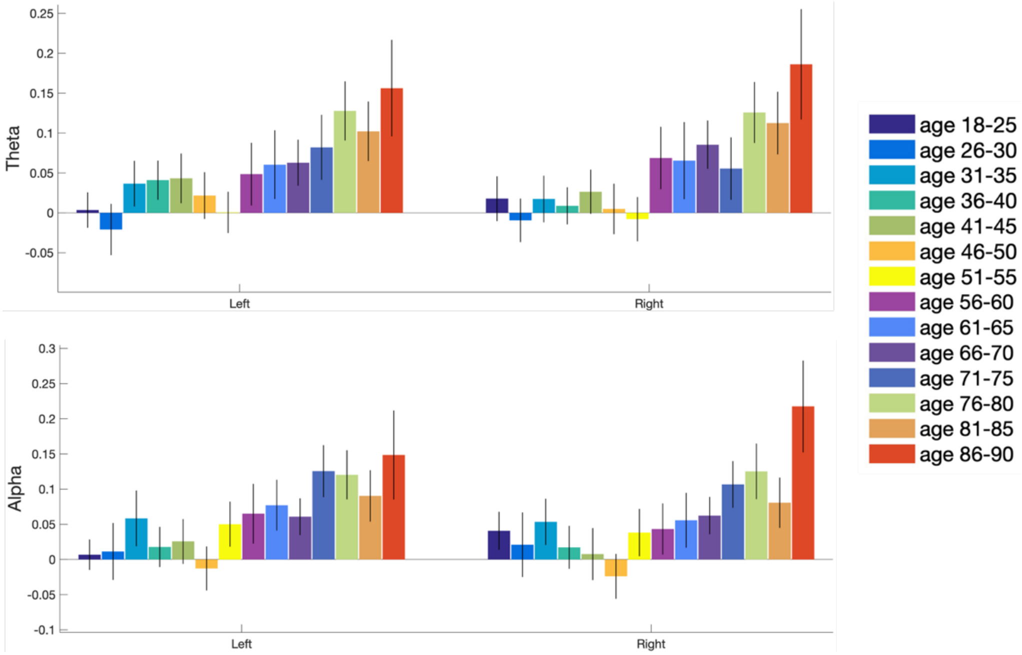

Figure S2. BRO spectral effects in the theta and alpha bands within the left and right precuneus, after dividing participants into age groups by 5-year bins.

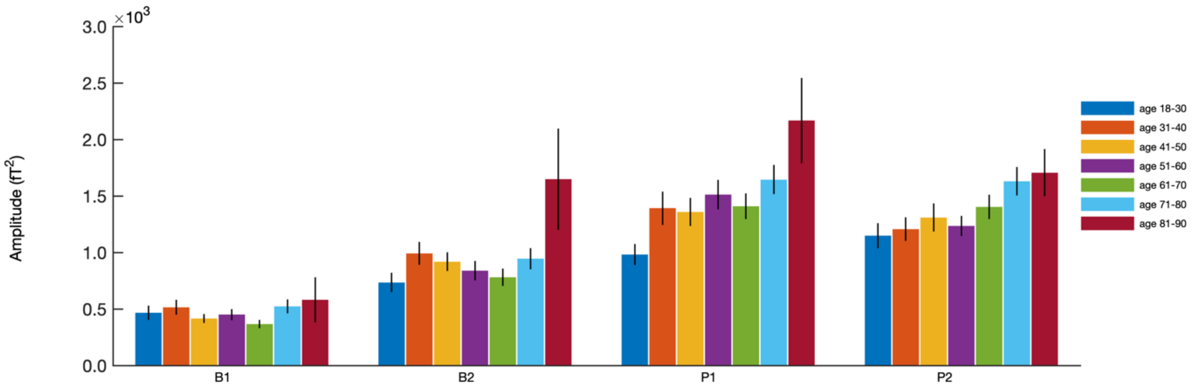

Figure S3. Sensor-level GFP amplitudes after dividing participants into age groups by 10-year bins.

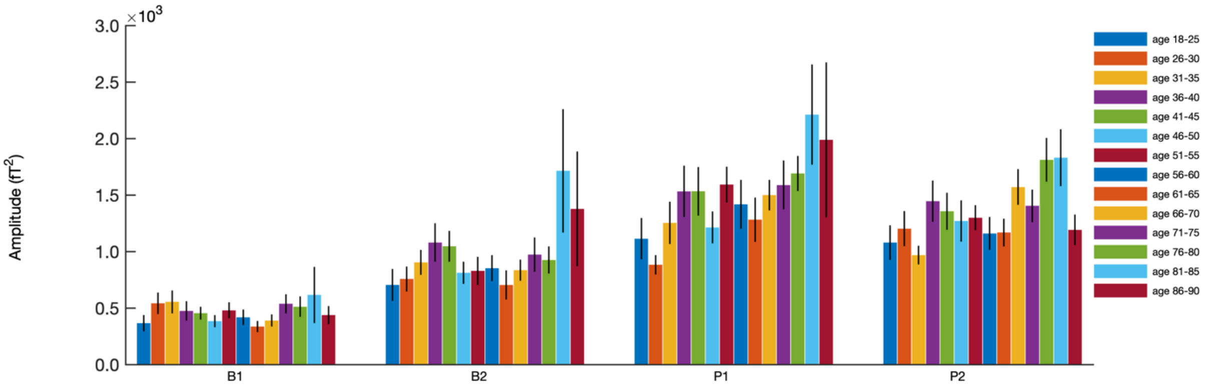

Figure S4. Sensor-level GFP amplitudes after dividing participants into age groups by 5-year bins.

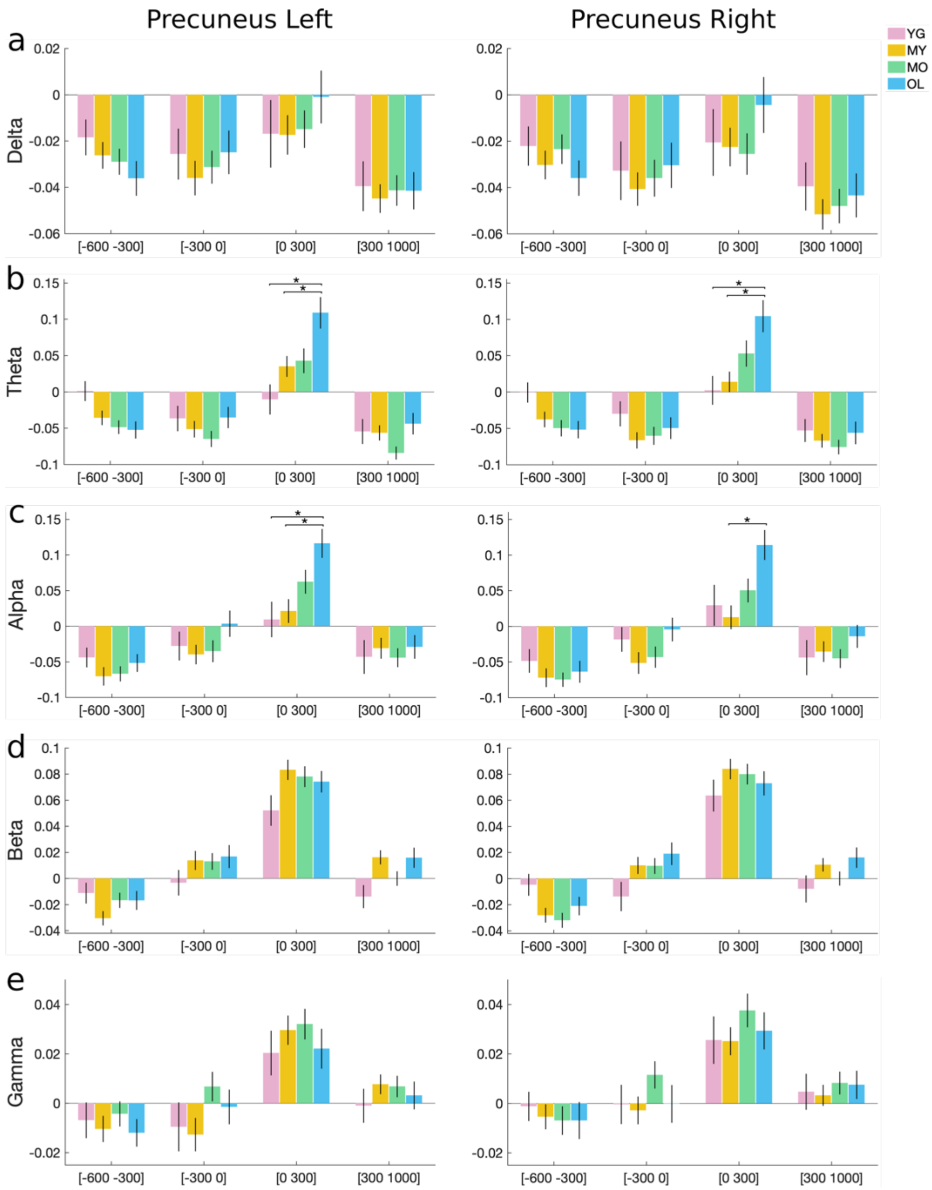

*Figure S5. BRO spectral effects within the bilateral precuneus, showing mean signal power in different frequency bands within different time intervals before and after blinking. Horizontal axis represents time in milliseconds. *p<0.05*
